# Supplementary material for: End Sequence Analysis Toolkit (ESAT) expands the extractable information from single-cell RNA-seq data
Source: Genome Res. 2016 Oct;26(10):1397–410. doi: 10.1101/gr.207902.116 (PMC5052061; doi:10.1101/gr.207902.116)
Supplement: Supplemental Material [file supp_gr.207902.116_Supp_Materials.tar.gz › ESAT-esat_v0.1/doc/html/class_jama_1_1_q_r_decomposition.html]

ESAT: Jama.QRDecomposition Class Reference


ESAT


- Main Page
- Related Pages
- Packages
- Classes
- Files

- Class List
- Class Index
- Class Hierarchy
- Class Members

All Classes Namespaces Files Functions Variables Enumerator Pages

- Jama
- QRDecomposition

Public Member Functions |
List of all members

Jama.QRDecomposition Class Reference

Inheritance diagram for Jama.QRDecomposition:

[legend]

Collaboration diagram for Jama.QRDecomposition:

[legend]

|  |  |
| --- | --- |
| Public Member Functions | |
|  | QRDecomposition (Matrix A) |
|  | |
| boolean | isFullRank () |
|  | |
| Matrix | getH () |
|  | |
| Matrix | getR () |
|  | |
| Matrix | getQ () |
|  | |
| Matrix | solve (Matrix B) |
|  | |

## Detailed Description

QR Decomposition.

For an m-by-n matrix A with m >= n, the QR decomposition is an m-by-n orthogonal matrix Q and an n-by-n upper triangular matrix R so that A = Q\*R.

The QR decompostion always exists, even if the matrix does not have full rank, so the constructor will never fail. The primary use of the QR decomposition is in the least squares solution of nonsquare systems of simultaneous linear equations. This will fail if isFullRank() returns false.

## Constructor & Destructor Documentation

|  |  |  |  |  |  |
| --- | --- | --- | --- | --- | --- |
| Jama.QRDecomposition.QRDecomposition | ( | Matrix | *A* | ) |  |

QR Decomposition, computed by Householder reflections.

Parameters
:   |  |  |
    | --- | --- |
    | A | Rectangular matrix |

Returns
:   Structure to access R and the Householder vectors and compute Q.

## Member Function Documentation

|  |  |  |  |  |
| --- | --- | --- | --- | --- |
| Matrix Jama.QRDecomposition.getH | ( |  | ) |  |

Return the Householder vectors

Returns
:   Lower trapezoidal matrix whose columns define the reflections

|  |  |  |  |  |
| --- | --- | --- | --- | --- |
| Matrix Jama.QRDecomposition.getQ | ( |  | ) |  |

Generate and return the (economy-sized) orthogonal factor

Returns
:   Q

|  |  |  |  |  |
| --- | --- | --- | --- | --- |
| Matrix Jama.QRDecomposition.getR | ( |  | ) |  |

Return the upper triangular factor

Returns
:   R

|  |  |  |  |  |
| --- | --- | --- | --- | --- |
| boolean Jama.QRDecomposition.isFullRank | ( |  | ) |  |

Is the matrix full rank?

Returns
:   true if R, and hence A, has full rank.

Here is the caller graph for this function:

|  |  |  |  |  |  |
| --- | --- | --- | --- | --- | --- |
| Matrix Jama.QRDecomposition.solve | ( | Matrix | *B* | ) |  |

Least squares solution of A\*X = B

Parameters
:   |  |  |
    | --- | --- |
    | B | A Matrix with as many rows as A and any number of columns. |

Returns
:   X that minimizes the two norm of Q\*R\*X-B.

Exceptions
:   |  |  |
    | --- | --- |
    | IllegalArgumentException | Matrix row dimensions must agree. |
    | RuntimeException | Matrix is rank deficient. |

Here is the call graph for this function:

---

The documentation for this class was generated from the following file:

- java/Jama/QRDecomposition.java


---

Generated on Fri Aug 15 2014 15:11:06 for ESAT by  

 1.8.7
